# Mapping Research Trends on Intestinal Permeability in Irritable Bowel Syndrome with a Focus on Nutrition: A Bibliometric Analysis

**DOI:** 10.3390/nu17061064

**Published:** 2025-03-18

**Authors:** Domenica Mallardi, Fatima Maqoud, Davide Guido, Michelangelo Aloisio, Michele Linsalata, Francesco Russo

**Affiliations:** 1 Functional Gastrointestinal Disorders Research Group, National Institute of Gastroenterology-IRCCS “Saverio de Bellis”, 70013 Castellana Grotte, Italy; domenica.mallardi@irccsdebellis.it (D.M.); fatima.maqoud@irccsdebellis.it (F.M.); michelangelo.aloisio@irccsdebellis.it (M.A.); michele.linsalata@irccsdebellis.it (M.L.); 2 Data Science Unit, National Institute of Gastroenterology-IRCCS “Saverio de Bellis”, 70013 Castellana Grotte, Italy; davide.guido@irccsdebellis.it

**Keywords:** bibliometric analysis, intestinal permeability, irritable bowel syndrome, gut microbiota, nutrition, ASReview, probiotics, machine learning

## Abstract

Irritable Bowel Syndrome (IBS) is a complex gastrointestinal disorder characterized by chronic abdominal pain and altered bowel habits, often linked to disruptions in intestinal barrier function. Increased intestinal permeability plays a key role in IBS pathogenesis, affecting immune responses, gut microbiota, and inflammation. This study conducts a bibliometric analysis to explore global research trends on intestinal permeability in IBS, focusing on key contributors, collaboration networks, and thematic shifts, particularly the interplay between the intestinal barrier, gut microbiota, and dietary components. A total of 411 articles were retrieved from Scopus, with 232 studies analyzed using Bibliometrix in R. To optimize screening, ASReview, a machine learning tool, was employed, utilizing the Naïve Bayes algorithm combined with Term Frequency-Inverse Document Frequency (TF-IDF) for adaptive ranking of articles by relevance. This approach significantly improved screening step efficacy. The analysis highlights growing research interest, with China and the USA as leading contributors. Key themes include the role of gut microbiota in modulating permeability, the impact of dietary components (fiber, probiotics, bioactive compounds) on tight junction integrity, and the exploration of therapeutic agents. Emerging studies suggest integrating gut barrier modulation with nutritional and microbiome-targeted strategies for IBS management. This study provides a comprehensive overview of research on intestinal permeability in IBS, mapping its evolution and identifying major trends. By highlighting key contributors and thematic areas, it offers insights to guide future investigations into the interplay between gut permeability, diet, and microbiota, advancing understanding of IBS pathophysiology and management.

## 1. Introduction

Irritable Bowel Syndrome (IBS) is a prevalent gastrointestinal disorder characterized by chronic abdominal pain, bloating, and altered bowel habits, without identifiable organic causes [1]. Despite its significant impact on quality of life, IBS’s underlying mechanisms and etiology remain poorly understood [2]. Recently, there has been growing interest in intestinal permeability as a key factor in IBS pathogenesis [3,4,5].

The intestinal barrier, a complex and dynamic structure, is critical in protecting the body from pathogens, toxins, and antigens in the intestinal lumen while enabling nutrient and water absorption [6,7]. This barrier comprises four main components: intestinal mucus, the epithelial layer, the mucosal immune system, and the gut microbiota. Among these, the intestinal epithelium is particularly important, with tight and adherens junctions forming a physical barrier regulating molecular passage [8]. Tight junctions, composed of transmembrane proteins such as claudins and occludins and cytoplasmic plaque proteins like zonula occludens, maintain barrier integrity and control permeability [9,10].

Increased intestinal permeability, often referred to as “leaky gut”, reflects compromised barrier function, allowing harmful substances to enter systemic circulation [11]. This phenomenon is closely linked to local inflammation, alterations in gut microbiota composition, and immune dysregulation, all contributing to IBS symptomatology [12,13].

To date, several scientific evidences highlight the intricate relationship between nutrition and intestinal permeability, a key regulator of intestinal barrier function and systemic health. Dietary components, such as fiber, polyphenols, and omega-3 fatty acids, strengthen tight junction integrity, reduce paracellular permeability, and mitigate luminal antigens’ translocation [14]. For instance, dietary fiber, particularly fermentable fibers like inulin and pectin, has been shown to improve barrier integrity by improving gut microbiota composition and enhancing SCFA production, which supports epithelial cell function and tight junction protein expression [15]. In contrast, excessive intake of ultra-processed foods, emulsifiers, and high-fat diets has been linked to increased intestinal permeability, promoting low-grade inflammation and metabolic dysfunction. For example, high-fat diets (HFDs) have been shown to alter tight junction proteins, increase gut permeability, and promote endotoxemia, which can lead to systemic inflammation and metabolic disorders [15]. In addition, the gut microbiota also plays a critical role in this dynamic, modulating epithelial responses through microbial metabolites such as short-chain fatty acids. Understanding these nutritional influences on intestinal barrier function offers new therapeutic avenues for conditions ranging from inflammatory bowel disease to metabolic syndrome [16].

The investigation of the intestinal barrier and its permeability mostly relies on non-invasive methods, mainly through quantifying urinary markers. The Lactulose/Mannitol (La/Ma) ratio is commonly used to assess small intestinal permeability, while urinary sucrose (Su) levels reflect gastroduodenal permeability [17,18,19]. Various circulating biomarkers are analyzed to gain deeper insights into intestinal permeability and barrier integrity. These include serum and fecal zonulin, a protein that regulates tight junctions and is often used as a marker of intestinal permeability [20]; serum intestinal fatty acid-binding protein (I-FABP), a marker of enterocyte damage that reflects intestinal epithelial injury [20]; and serum diamine oxidase (DAO), an enzyme produced by enterocytes whose levels correlate with mucosal integrity and are reduced in conditions of intestinal barrier dysfunction [20,21].

To develop a thorough understanding of this field, bibliometric analysis may serve as an effective tool for assessing trends in the scientific literature, identifying influential studies, and mapping research collaborations [22,23,24]. By analyzing large datasets of academic articles, bibliometric analysis can identify the most influential studies, key research topics, and emerging areas of interest over time. This method uses quantitative measures, such as citation counts and publication frequencies, to gauge the impact and relevance of specific works, authors, journals, and institutions. Moreover, bibliometric analysis provides insights into the structure of scientific networks by revealing collaboration patterns among researchers and institutions across different regions and disciplines. It helps identify leading contributors and influential groups driving advancements in a field and the geographical distribution of research activities [25,26].

To enhance the efficiency and accuracy of the literature selection process, this study employed ASReview, a machine learning-based tool, to expedite identifying and screening relevant articles. This approach streamlined the workflow and ensured a comprehensive and unbiased selection of studies for analysis [27].

This study aims to conduct the first bibliometric analysis focusing on the impact of intestinal permeability in IBS, emphasizing the intricate interactions between intestinal permeability, gut microbiota, and dietary factors. Using data from 1984 to July 2024, we aim to elucidate the evolution of research in this area, identify knowledge gaps, and highlight promising directions for future investigation.

## 2. Materials and Methods

### 2.1. Research Criteria and Record Identification

A bibliometric analysis was conducted using a dataset from the Scopus database, which is widely recognized for its extensive use in academic and bibliometric research [28,29,30,31]. Scopus offers comprehensive coverage across diverse scholarly fields and provides accurate citation statistics, enabling detailed assessments of topic development and trend shifts [32].

**Figure 1:**
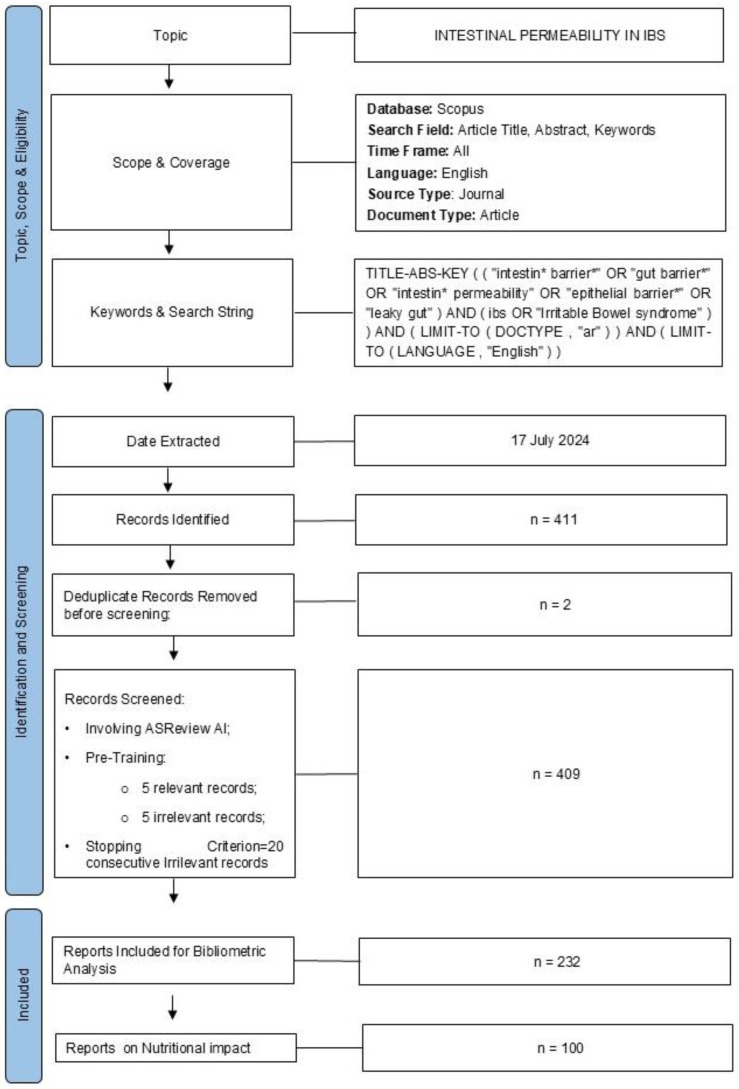
Flowchart of literature screening. Diagrammatic illustration of the methodology for gathering data and structuring the study.

Specific criteria were established to extract relevant records, including the search query, publication timeframe, language, and document type. The search query focused on two key terms: “irritable bowel syndrome” and “gut barrier”. The complete query was: TITLE-ABS-KEY ((“intestin* barrier*” OR “gut barrier*” OR “intestin* permeability” OR “epithelial barrier*” OR “leaky gut”) AND (IBS OR “Irritable Bowel Syndrome”)) AND (LIMIT-TO(DOCTYPE, “ar”)) AND (LIMIT-TO(LANGUAGE, “English”)) (Figure 1). The publication period was set to include all relevant articles within the database, confined to English-language publications. This analysis involved only original research articles, by excluding reviews.

The retrieved records were saved in two formats: Research Information Systems (RIS) and Comma Separated Values (CSV) files, both of which are available on GitHub (https://github.com/MichelangeloAloisio/Bibliometric_Analysis_IBS_permeability/, accessed on 3 March 2025). Each record in these files includes citation details, bibliographic information, abstracts, keywords, funding specifics, and additional data.

### 2.2. Record Screening

The screening process was conducted using ASReview (version 1.6.2) [27], a tool that integrates human expertise with artificial intelligence through an active machine learning (ML) “researcher-in-the-loop” algorithm. ASReview was used for screening at the title and abstract level, and the selected articles were subsequently analyzed using bibliometric software. This approach enhances the screening process’s speed and efficiency, enabling a more targeted and focused review [27].

Firstly, in a pre-processing step, the dataset RIS was examined for removing duplicate records (Figure 1).

The first step of the process was performed by machine learning and considered a “prior knowledge” phase, involving a pre-training setting where five relevant and five irrelevant articles were randomly selected [33,34,35,36,37,38,39,40,41]. Subsequently, the second ML-based step, representing the screening process core, was carried out using the Naïve Bayes (NB) algorithm combined with Term Frequency-Inverse Document Frequency (TF-IDF). This strategy was used in the active training phase for its superiority over other algorithms [26,42,43]. Specifically, the NB classifier uses TF-IDF to evaluate the relevance of an article by emphasizing the unique contribution of individual words within the entire corpus relative to the number of articles in which those words appear 38. Records were presented iteratively to human reviewers during this step, ordered by decreasing relevance to the bibliometric topic. In this case, records that quantitatively or qualitatively assessed intestinal permeability in IBS, irrespective of the technique, biomolecule used, or results obtained, were manually classified as appropriate for bibliometric analysis. The screening process continued until the identification of 20 consecutive irrelevant articles (i.e., stopping criterion). The studies encompassed experiments conducted across various platforms “in vitro, ex vivo, and in vivo” without discrimination based on the species studied. The screening process was expanded to include a full-text review for records with ambiguous titles and abstracts. During this process, review records misclassified as original articles in the Scopus database were identified and removed. In the same way, documents not written in English or lacking an abstract were removed.

The final dataset, comprising only relevant records, is available in RIS format at the following GitHub link: https://github.com/MichelangeloAloisio/Bibliometric_Analysis_IBS_permeability/ (accessed on 3 March 2025).

### 2.3. Bibliometric Analysis

The bibliometric analysis was conducted using the Bibliometrix and rworldmap R packages [25] with additional support from the user-friendly Biblioshiny web interface. This package employs various statistical and mathematical methods to evaluate and analyze datasets.

The bibliometric analysis provided several indexes and plots to explore the scientific topic of intestinal permeability in IBS from a bibliometric perspective. Overall, the number of published articles and citations per year and the (co)occurrence of authorships and affiliations (i.e., collaboration analysis) were revealed. In addition, the occurrence of journals and author keywords was also analyzed.

Firstly, a ‘Publications per Year’ plot was derived to analyze the number of publications per year and the total number of citations to assess the research’s impact over time.

Thus, a “Country Scientific Production” plot was also generated to illustrate the global distribution of publications on the relationship between IBS and intestinal permeability.

The ‘Sources Production Over Time’ analysis examined journal production cumulatively, using the ‘Occurrences’ index to track the cumulative count of journal appearances in the dataset. The study focused on the top 10 journals to highlight the most prolific sources in the field.

To analyze “authors’ production over time”, metrics such as the annual number of publications and total citations received were used. The analysis focused on the top 25 most prolific authors. This approach was chosen to highlight publication trends and the impact of leading contributors by examining the occurrences and cumulative metrics of their output.

The authors also performed a “Collaboration Networks analysis”. For this, co-authorship connections were evaluated, where nodes represented authors and edges indicated shared publications. Key metrics such as node degree and betweenness centrality were calculated to identify influential authors and collaboration patterns. Community detection algorithms were applied to reveal clusters of frequent collaborators. The network was visualized using a force-directed layout to effectively display the structure and intensity of collaborative relationships. The set parameters included 50 nodes, a repulsion force of 0.1, at least 3 edges, and the exclusion of isolated nodes, with default graphical settings applied.

The “Most Relevant Affiliations” analysis identified the institutions most frequently associated with the publications in the dataset. Affiliation data were extracted and aggregated to determine the institutions with the most contributions. This analysis provided insights into the geographic and institutional distribution of research output, highlighting the key contributors to the field. The data were extracted for the “Most Relevant Affiliations” figure by enabling the “Affiliation Name Disambiguation” option and setting the Number of Affiliations to 10.

Thus, a “Collaboration Network” analysis was performed on countries employing the Walktrap clustering algorithm, with the automatic network layout and association as the normalization type. The parameters set included 50 nodes, a repulsion force of 0.1, a minimum of 1 edge, and the exclusion of isolated nodes, while default graphical settings were used. In the network map, nodes represent countries, with their sizes proportional to the number of publications. The thickness of the lines connecting nodes indicates the strength of the co-authorship relationships. Moreover, clusters highlighted in different colors represent countries with similar collaboration patterns.

Subsequently, a text analysis was applied to the author’s keywords. A “Word Cloud” Plot visually represents the frequency of author keywords, with the size of each keyword indicating its prominence within the dataset. This plot was generated using 100 author keywords, excluding those removed during the Scopus dataset extraction.

Trends and shifts in focus across different journals were evaluated to analyze the evolution of research topics over the dataset’s timespan. This approach used default parameters to assess how topics developed throughout the study period, providing a comprehensive overview of topic progression, as illustrated by the ‘Trend Topic’ analysis.

Finally, a “Three-Field” analysis explored and illustrated the relationships between three key bibliometric fields: authors, keywords, and countries. The relative plot effectively maps the connections between the most prolific authors, the most frequently occurring keywords, and the countries of the affiliated institutions, offering a comprehensive overview of the research dynamics within the dataset. In this analysis, the “Three-Field” Plot was generated by selecting 20 items for each field (authors on the left, keywords in the middle, and countries on the right).

All datasets, R scripts, and keyword lists used for this analysis are available on the GitHub repository at https://github.com/MichelangeloAloisio/Bibliometric_Analysis_IBS_permeability/ (accessed on 3 March 2025).

### 2.4. Nutritional Impact Analysis

To identify records concerning the impact of nutrition on intestinal permeability in IBS, we selected relevant records from the final dataset, which contained the relevant entries identified by ASReview (version 1.6.2) [27]. Specifically, we used a Python (version 3.10.9) script, available on the GitHub repository at https://github.com/MichelangeloAloisio/Bibliometric_Analysis_IBS_permeability/, to filter records related to the role of nutrition. This script included only those records that contained the keywords ‘nutrition’, ‘diet’, ‘food’, ‘nutrient’, ‘microbiome’, ‘gut microbiota’, ‘probiotic’, or ‘prebiotic’ in the title, abstract, or keyword section. Subsequently, these filtered records were manually reviewed.

## 3. Results

### 3.1. Dataset Download and Screening

**Figure 2:**
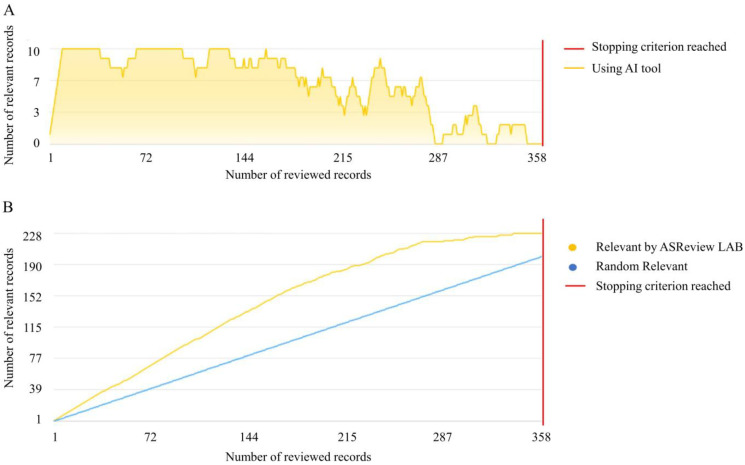
Active Machine Learning Process. This figure features two charts, the Progress Chart (**A**) and the Recall Chart (**B**), to monitor the dynamic progression of an active machine-learning process. A critical threshold, indicated by the red line, marks the stopping criterion. (**A**) Progress Chart: This shows the total count of relevant records spotted in the last 10 manually checked entries, indicating the model’s learning pace over time (https://asreview.readthedocs.io/en/stable/progress.html, accessed on 30 July 2024). (**B**) Recall Chart: Compares the ASReview-enhanced ranking system’s performance against a randomly arranged baseline, underscoring the ASReview system’s enhanced ability to pinpoint relevant records beyond random selection (https://asreview.readthedocs.io/en/stable/progress.html, accessed on 30 July 2024).

Using the comprehensive query in Figure 1, 411 records were initially extracted from the Scopus database. Before beginning the screening process, 2 duplicate entries were identified and removed, leaving 409 records for further analysis (Figure 1). These remaining records were then screened using the ASReview active learning system. In addition to the 10 articles reviewed during the ‘prior knowledge’ step, 358 were manually evaluated (Figure 2A,B). Instead, 41 articles remained unreviewed.

The screening phase concluded upon reaching the predefined stopping criterion (Figure 2A,B). This process identified 232 relevant records (Figure 2), forming the basis for subsequent bibliometric analysis. Conversely, 136 records were deemed irrelevant. Employing the ASReview tool on the Scopus dataset minimized the need for manual review to 89% of the dataset, effectively reducing the workload on human reviewers by 11%.

### 3.2. Dataset General Description

The bibliometric analysis was conducted on 232 publications produced between 1984 and 2024. The annual average growth rate, calculated using bibliometric tools, was 7%, indicating a significant increase in publication output over the years. From 1984 to 2009, fewer than five articles were published annually. However, between 2010 and 2018, this number rose to between five and 15 articles per year. Notably, since 2019, the annual publication count has consistently exceeded 15 articles. This trend highlights a growing research focus on evaluating intestinal permeability in IBS over the years.

**Figure 3:**
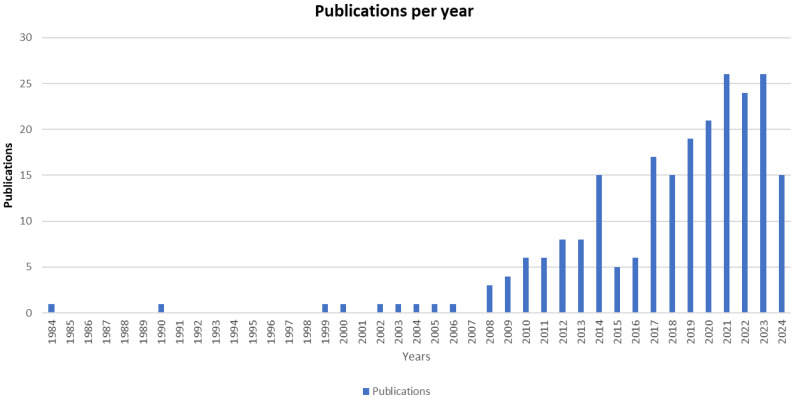
Publications per year. The evolution in research publications regarding the connection between IBS and permeability can be traced through the rising count of such publications.

Both Figure 3 and Table 1 offer a comprehensive overview of these studies, including their citation numbers, which reflect their influence and importance within the scientific community. A high citation rate generally implies that a paper has significantly advanced the discipline, given its widespread use as a reference by other researchers.

### 3.3. Geographical Distribution and International Collaboration Network

**Figure 4:**
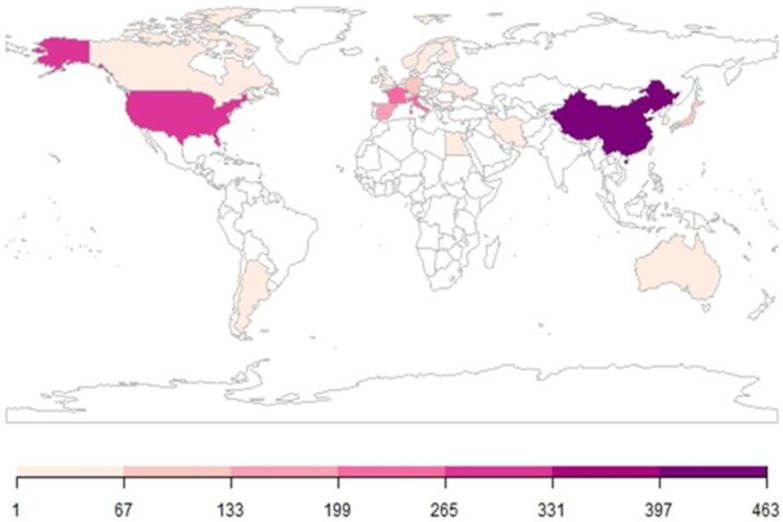
Country scientific production plot. The global distribution of publications concerning the relationship between IBS and intestinal permeability. Countries without contributions in this field are marked in white. The legend shows the number of authors’ appearances by country affiliations.

Twenty-seven global nations have contributed to publications on IBS and intestinal permeability. The top 10 contributing countries are China (463; 23.7%), the United States (273; 14.0%), France (251; 12.8%), Italy (246; 12.6%), Spain (170; 8.7%), Japan (116; 5.9%), Germany (83; 4.2%), South Korea (60; 3.1%), Canada (56; 2.9%), and Sweden (54; 2.8%), with China and the USA taking the lead (Figure 4). The results show a broad global interest and effort in this field. Moreover, China, the United States, France, and Italy emerged as the most significant contributors, producing more than 60 percent of the total publications. This suggests that these countries are probably investing heavily in this area of research.

**Figure 5:**
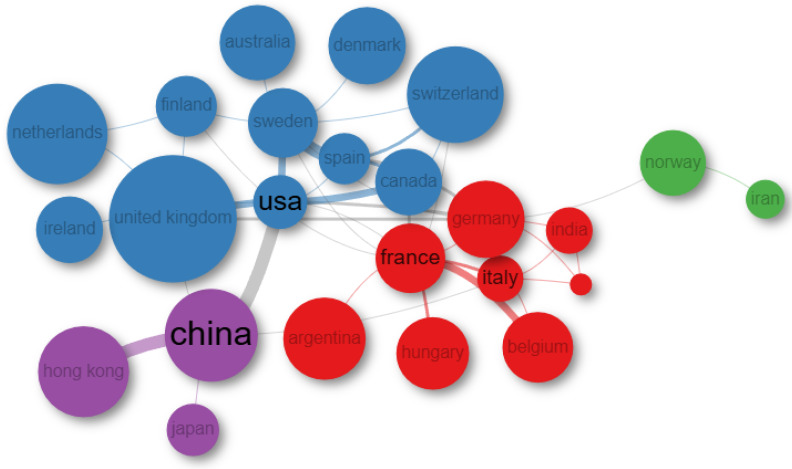
Collaboration network by country. Map of international collaboration networks in Intestinal Permeability and IBS. The minimum number of edges threshold was set equal to 1.

The map in Figure 5 illustrates the collaboration between countries in scientific research on gut permeability and IBS. The map reveals four distinct clusters, each highlighted in different colors, representing countries with similar collaboration patterns.

China and the USA emerged as the primary hubs in international research, showcasing the most frequent collaborations. The USA has notably strong partnerships with the UK, Canada, Spain, and Sweden. China also maintains significant collaborations with Japan and Hong Kong.

Another cluster, predominantly European, includes Argentina as an extra-EU country. Within this cluster, the main collaborations occur between France and Belgium. Additionally, Norway and Iran have an isolated network of cooperation.

### 3.4. Most Productive Journals

**Figure 6:**
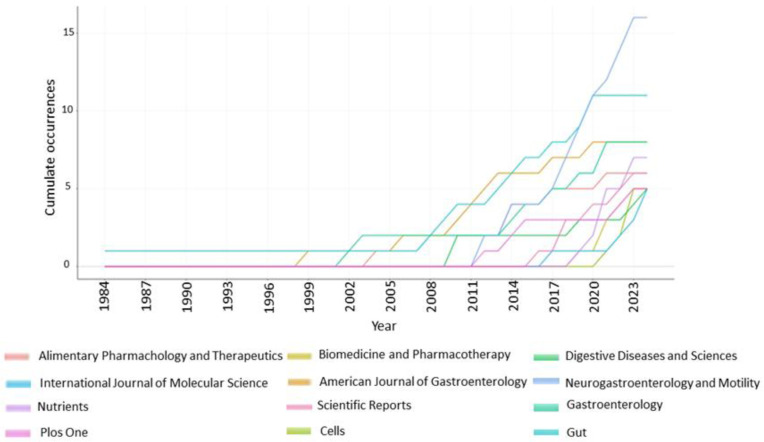
Sources production over time. The most cumulative productive journals (more than 10 published papers).

Figure 6 highlights the 10 most prolific journals, each publishing over 10 articles. The top five include Neurogastroenterology and Motility, Gut, American Journal of Gastroenterology, and Nutrients. The analysis reveals a significant increase in journals covering the topic since 2011.

### 3.5. Authors and Institutions

**Figure 7:**
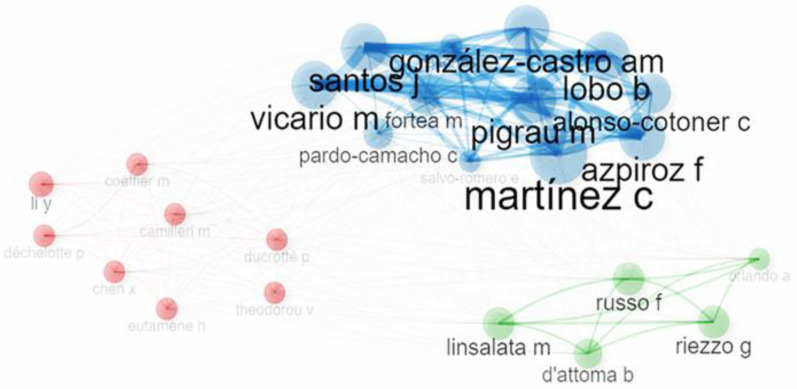
Collaboration networks by authors. Networks and the existing citations among the top 25 authors of the topic. The minimum number of edges threshold was set equal to 3.

A total of 1383 authors contributed to 232 documents. Figure 7 illustrates three distinct clusters, where the nodes within each cluster exhibit frequent communication and similar research trajectories. It is worth pointing out that although the clusters are differentiated by color, they are not entirely isolated and display moderate interconnection.

**Figure 8:**
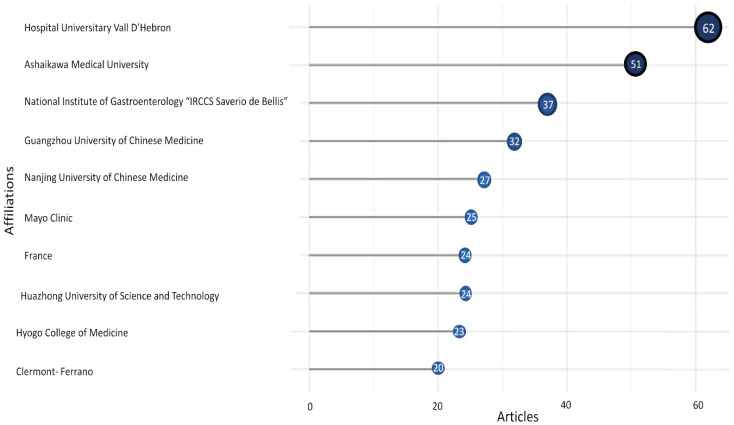
Most Relevant Affiliations. The 10 most prolific organizations on IBS and Intestinal Permeability.

Figure 8 illustrates the top 10 prolific organizations with 25 or more published documents. Leading the ranking are “Vall d’Hebron University Hospital” with 62 publications, “Asahikawa Medical University” with 51 publications, and the “National Institute of Gastroenterology IRCCS Saverio De Bellis” with 37 publications.

### 3.6. Most Global Cited Documents

The top 10 most globally cited articles on intestinal permeability in IBS are listed in Table 2. The highest number of citations (*n* = 454) was found in the article “Impaired intestinal barrier integrity in the colon of patients with irritable bowel syndrome: involvement of soluble mediators”, published in 2009 in “Gut” which is one of the high-impact scientific journals in the field of gastroenterology. The most-cited articles (number of citations = [233–454]) were published between 2002 and 2017, suggesting that this period has enormously contributed to the scientific understanding in this area. Three articles on this top list were published in journals with an impact factor greater than 10, indicating the influence of these works in the scientific community.

### 3.7. Three-Fields Plot

The three-field plot visually represents the relationships between various parameters, including sources, countries, affiliations, keywords, leading authors, cited sources, and author keywords. In these rectangular diagrams, different colors represent pertinent elements, and the size of each rectangle correlates with the number of relationships among the selected components.

**Figure 9:**
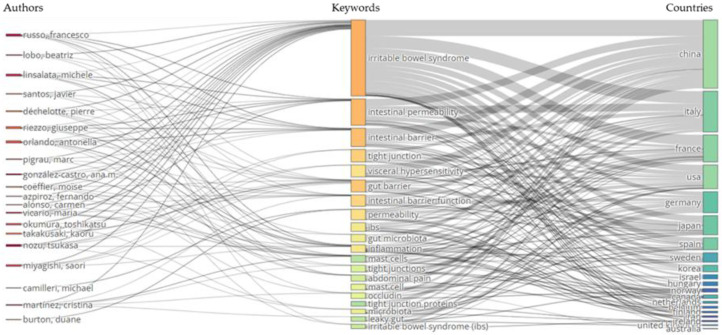
The three-field plot of the networks between authors (**left**), keywords (**middle**), and countries (**right**) of articles on intestinal permeability in IBS.

Figure 9 illustrates the plot for research on intestinal permeability in IBS, highlighting the connections between authors (left), author keywords (middle), and countries (right). Analysis of the top keywords, authors, and countries reveals that terms such as “irritable bowel syndrome”, “intestinal permeability”, and “intestinal barrier” are frequently used. Notably, these keywords are prominently featured in the work of leading authors, including Russo F., Nozu T., and Okumura T.

### 3.8. Word Cloud Frequency

**Figure 10:**
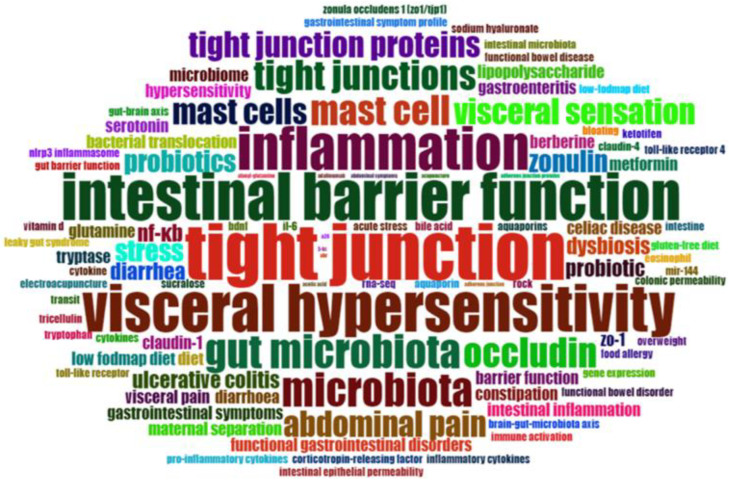
Word cloud plot. The most common author keywords on IBS and Intestinal Permeability. The size of each word reflects its frequency and significance within the database.

The keyword analysis aimed to identify the most commonly used author keywords within the extracted dataset, excluding terms originally included in the Scopus research query. The list of removed words is available on GitHub [https://github.com/MichelangeloAloisio/Bibliometric_Analysis_IBS_permeability/, accessed on 3 March 2025]. The results of this analysis are summarized in the word cloud plot (Figure 10), which visually represents the most frequently occurring author keywords associated with irritable bowel syndrome (IBS) and intestinal permeability. In the word cloud, the size of each word reflects its prevalence and significance within the dataset: larger words correspond to widely discussed topics, while smaller words indicate less-explored areas that may warrant further investigation.

The analysis revealed that the most widely discussed topics related to IBS and intestinal permeability include “tight junction”, “intestinal barrier function”, “visceral hypersensitivity”, “inflammation”, “gut microbiota”, “dysbiosis”, “mast cell”, “occludin”, “probiotics”, “stress”, and “zonulin”. In contrast, less frequently addressed topics, which represent potential areas for future research, include “alanyl-glutamine”, “adalimumab”, “acupuncture”, “acetic acid”, “5-HT”, “Vitamin D”, “tryptophan”, “serotonin”, “tricellulin”, “Toll-like receptor”, “NLRP3 inflammasome”, “cytokines”, “miR-144”, “low-FODMAP diet”, “food allergy”, “eosinophil”, “BDNF”, “aquaporin”, and “claudin”.

### 3.9. Trending Topics About Intestinal Permeability in IBS

**Figure 11:**
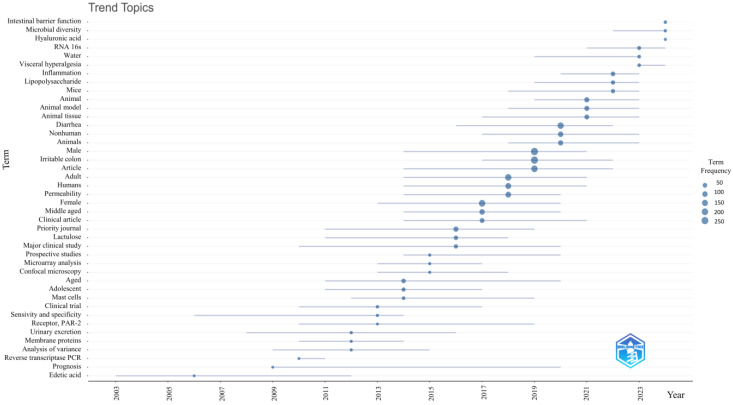
Trend topics evolution during 2003–2024. The lines in the graph show how topics have been dynamically changing and trending in different years. The earlier the line appears, the earlier the topic appears in the field of study. The dots on the lines represent the number of occurrences of the IBS and Intestinal Permeability, with larger circles indicating more occurrences.

The evolution of core keywords over time is illustrated in Figure 11. Analysis shows that between 2020 and 2021, the most frequently repeated KeyWords Plus included terms like “animal”, “animal model”, “animal tissue”, and “nonhuman”. This suggests that research during this period was primarily focused on preclinical studies involving animal models. In 2022, the primary keywords shifted to “inflammation” and “lipopolysaccharide”, indicating increased ion to the relationship between bacterial lipopolysaccharide, its inflammatory effects, and gut barrier dysfunction.

The temporal trend analysis further reveals that recent hotspots include keywords such as “intestinal barrier function”, “microbial diversity”, “hyaluronic acids”, and “visceral hyperalgesia”. Current research focuses on the impact of microbial diversity on intestinal barrier function and exploring dietary components to alleviate IBS symptoms. These emerging research directions are likely to continue evolving in the future.

### 3.10. Nutritional Impact Results

A total of 100 articles, representing 43% of the total articles included in the final dataset containing the relevant records identified by ASReview (version 1.6.2) [27], discussed the impact of nutrition on intestinal permeability in IBS. The file containing the articles is available at the link: https://github.com/MichelangeloAloisio/Bibliometric_Analysis_IBS_permeability/ (accessed on 3 March 2025). These articles were produced by 547 authors, accounting for 39.5% of the total number of authors addressing IBS and permeability in general. Among the 10 most globally cited works (Table 2), 30% discuss the relationship between IBS, permeability, and nutrition [46,49,53]. The keyword co-occurrence analysis (Figure 10) revealed the most widely discussed topics related to nutrition, such as “gut microbiota”, “dysbiosis”, and “probiotics”, as well as less explored topics that represent potential areas for future research. The temporal trend analysis (Figure 11) on nutrition further reveals that recent hotspots include keywords such as “microbial diversity” and “hyaluronic acids”.

## 4. Discussion

This study presents the first bibliometric analysis focusing on the impact of intestinal permeability in IBS, with a particular emphasis on the complex interactions between intestinal permeability, gut microbiota, and dietary factors. A novel aspect of this research is the initial selection of 411 articles from Scopus [28], followed by screening using the ‘ASReview’ tool, which employs active machine learning to automate and enhance the process. This method not only reduces researchers’ workload but also prioritizes the most relevant studies, continuously refining its accuracy through feedback during the review of a small subset of articles [27]. The analysis spans from 1984 to July 2024 and was performed using the bibliometrix package and the biblioshiny web interface [25].

As a result, 232 original articles have been published over the past 40 years, reflecting the evolving interest in this field. The growing recognition of the intestinal barrier’s role in IBS is evident from the sharp increase in annual publications. Initially, the field experienced a modest annual growth rate of 0.4% until 2006, followed by notable increases to 1.3%, 1.7%, and 2.6% in 2008, 2009, and 2010, respectively. A significant surge occurred after 2014, with a 6.5% increase in scientific output, likely driven by increased research funding and the pressures of academic publishing (Figure 3, Table 1). Parallel to the rise in publications, citations in this field have also grown over time, and the top 10 cited articles are detailed (Table 2). The period between 2009 and 2019 saw the highest citation rates, peaking in 2014 (Figure 3). This suggests that studies published during this period have a substantial impact on the field, as they are frequently referenced by other researchers, highlighting their influence on advancing the discipline.

The global distribution of research on the link between IBS and intestinal permeability underscores widespread international engagement. Figure 4, which maps country-specific contributions, reveals that the most active countries are primarily concentrated in Europe, Asia, and North America. This geographic concentration of research activity suggests that these regions are heavily investing in this area, reflecting the growing acknowledgment of intestinal permeability as a critical factor in IBS pathophysiology. The growing recognition of the intestinal barrier’s role in IBS is likely also influenced by the evolution of research funding structures across different geographical regions. Funding ecosystems, which consist of networks of funders, regulatory bodies, and supporting institutions, play a crucial role in determining which projects receive resources and how scientific challenges are addressed. For example, in the United States, the funding system is characterized by a combination of federal funding and a particularly well-developed private philanthropy sector, whereas the European Union adopts a more centralized approach, with programs aimed at scientific integration and addressing common challenges [54]. These structural differences may influence the distribution of research on IBS and intestinal permeability, shaping which countries emerge as leaders in scientific output on these topics.

Scientific collaborations generally enhance research productivity. Notably, cooperative research outcomes, especially those resulting from international partnerships, tend to be of higher scientific quality and achieve greater academic impact. Indeed, China, the United States, and France (Figure 5), in addition to being the most prolific contributors to IBS and intestinal permeability research, also demonstrate a strong tendency toward international collaboration, further reinforcing their influence in the field.

Among the 1384 international authors contributing to research on intestinal permeability in IBS, this significant proportion highlights the growing interest in the role of intestinal permeability and their potential implications for IBS pathophysiology and management. Moreover, the top contributors and collaborators (Figure 7), González-Castro A.M., Nozu T., Russo F., Vicario M., Linsalata M., Lobo B., Martínez C., Miyagishi S., Okumura T., and Orlando A. have made significant contributions to the field. According to the most active authors, the most relevant affiliations (Figure 8) are the “Vall d’Hebron University Hospital”, which conducted the highest number of publications, followed by “Asahikawa Medical University” and the “National Institute of Gastroenterology-S. de Bellis-Research Hospital”. Together, they contributed approximately 44.6% of the total publications, highlighting the global collaboration and institutional efforts in this field.

Keyword co-occurrence analysis (Figure 10) identified key areas of interest and emerging research topics. Major themes included “tight junctions”, “intestinal barrier function”, and “zonulin”, underscoring the importance of intercellular junctions in barrier integrity [55]. Additionally, the links between “visceral hypersensitivity”, “inflammation”, “mast cells”, and “stress” highlighted the complex interactions contributing to IBS symptomatology [56,57]. The role of the gut microbiota, particularly “probiotics” and “dysbiosis”, has gained traction, reinforcing the significance of nutritional and microbial modulation strategies in IBS management [56].

The keyword analysis also emphasized the necessity of further exploring specific topics related to intestinal permeability in IBS. In particular, the gut microbiota appears to play a significant role in the gut-brain axis, warranting additional investigation. This is reflected in keywords such as “Tryptophan”, “Serotonin”, “BDNF”, “5-HT”, “Toll-like receptor”, “vitamin D”, and “cytokines [58]. Overall, the increasing usage of specific terms, particularly from 2009 onward (Figure 11), reflects the growing interest in intestinal permeability research and its clinical implications. Since 2010, clinical and prospective studies assessing intestinal permeability in IBS patients using various techniques have surged, peaking in 2017. More recently, research has shifted toward preclinical studies in animal models, with a significant rise between 2020 and 2021 [6]. Over the last two years, investigations have primarily focused on the intricate interplay between bacterial LPS-induced inflammation, alterations in intestinal barrier function, and visceral pain [59]. These factors are not only pertinent to IBS but also to a broader range of gastrointestinal disorders.

Other underexplored topics, including “alanyl-glutamine”, “adalimumab”, “acupuncture”, and “low-FODMAP diet”, refer to dietary and pharmacological interventions aimed at alleviating visceral pain and IBS symptoms, highlighting the need for further studies in these areas.

Concerning the Nutritional Impact results, a total of 547 (39.5%) authors have focused on nutrition-related aspects. This significant proportion highlights the growing interest in the role of dietary factors in modulating intestinal permeability and their potential implications for IBS pathophysiology and management.

Given that China and the USA are major contributors to IBS and intestinal permeability research, dietary habits in these countries reflect distinct priorities shaped by cultural, socio-economic, and environmental factors. Indeed, while Chinese studies emphasize traditional diets (e.g., fermented foods, spicy/cold foods) and herbal remedies [60], US research focuses on addressing Western diet effects (e.g., high-fat, processed foods) through dietary modifications and pharmacological treatments [61]. These differences highlight culturally tailored approaches to managing IBS and underscore the importance of considering regional dietary patterns when interpreting research findings. Furthermore, they raise questions about the generalizability of nutritional interventions across populations with diverse dietary habits. Bridging these gaps may require international collaborations and standardized methodologies to ensure that research outcomes are applicable globally.

As outlined previously, key contributing authors, such as González-Castro A.M., in collaboration with Vicario M., Lobo B., and Martínez C., have demonstrated that grape seed extract, which is rich in polyphenols, reduces visceral hypersensitivity in IBS models by suppressing TLR4-cytokine signaling while preserving tight junction integrity [62].

Moreover, F. Russo, Linsalata M., and Orlando A. have also made substantial contributions to understanding irritable bowel syndrome with diarrhea predominance (IBS-D), particularly regarding intestinal barrier dysfunction, inflammation, and nutrition [30]. These authors have explored various dietary strategies to improve intestinal barrier function and alleviate symptoms. For instance, an extract of Lens culinaris, rich in flavonoids, was shown to protect the intestinal mucosa from LPS-induced inflammation, enhancing transepithelial electrical resistance and tight junction integrity while reducing inflammatory markers. This suggests a potential therapeutic role for flavonoid-rich extracts in gastrointestinal inflammatory diseases [63]. Similarly, a diet based on Tritordeum was found to significantly improve gastrointestinal symptoms and intestinal barrier health in IBS-D patients, reducing intestinal permeability and markers of dysbiosis and inflammation. This highlights the potential of this ancient cereal variety as a beneficial dietary intervention for IBS-D [64].

The authors also investigated the role of vitamin D in IBS-D, finding that patients with low vitamin D levels (L-VD) exhibited more severe symptoms and altered intestinal permeability compared to those with normal levels (N-VD). Interestingly, a Low FODMAP Diet (LFD) not only improved intestinal permeability but also increased vitamin D levels, suggesting a dual therapeutic effect that could alleviate both gastrointestinal and systemic symptoms [65]. Furthermore, a 12-week LFD was shown to significantly improve gastrointestinal symptoms, psychological well-being, and intestinal permeability, alongside reductions in inflammatory markers. These findings underscore the potential of dietary interventions to address both the clinical and psychological dimensions of IBS-D, aligning with the biopsychosocial model of the condition [64].

Among the 10 most cited papers (Table 2), 30% have explored the role of intestinal permeability and its interactions with the gut microbiota and dietary factors in IBS. These studies, published in high-impact journals (all Q1), have provided foundational evidence for understanding how intestinal barrier dysfunction contributes to IBS pathophysiology and how dietary interventions might modulate the gut microbiota [46].

One of the most significant contributions is the study by De Palma et al. (2017) [46], published in Science Translational Medicine (IF: 15.8, Q1), which demonstrated that fecal microbiota transplantation from IBS-D patients to germ-free mice could induce changes in gut function and behavior, including anxiety and intestinal barrier dysfunction. The study showed that mice receiving the IBS-D microbiota exhibited faster gastrointestinal transit, intestinal barrier dysfunction, innate immune activation, and anxiety-like behavior compared to those receiving microbiota from healthy controls. This suggests that microbiota modulation through nutritional or pharmacological interventions could be an effective strategy for managing IBS [53]. For instance, Xu et al. (2014) [53], published in Gastroenterology (IF: 25.7, Q1), explored the role of rifaximin, a non-absorbable antibiotic, in modulating the gut microbiota and preventing stress-induced gut inflammation and visceral hyperalgesia in rats. The results showed that rifaximin altered the microbiota composition, promoting the growth of *Lactobacillus*, and improved gut barrier function. Additionally, the study by Gecse et al. (2008) [49], published in Gut (IF: 23, Q1), highlighted the role of elevated colonic luminal serine protease activity in IBS-D. This increased protease activity impairs colonic paracellular permeability and induces visceral hypersensitivity through the activation of the PAR-2 receptor, suggesting potential novel dietary strategies targeting protease activity.

Moreover, the most recent research efforts have concentrated on enhancing intestinal barrier function in IBS patients through dietary interventions, gut microbiota modulation, and novel therapeutic strategies. A promising study investigated the administration of pasteurized Akkermansia muciniphila, a predominant symbiont in the human gut mucosa, in two mouse models of IBS with different etiologies. While A. muciniphila administration did not significantly alter gut microbiota composition, it effectively reduced colonic hypersensitivity, strengthened the intestinal barrier, and mitigated anxiety-like behaviors and cognitive impairments in post-infectious IBS (PI-IBS) models. These findings suggest that A. muciniphila, which is naturally enriched in fiber-rich diets, could offer a promising non-pharmacological approach for managing IBS-associated symptoms, including chronic abdominal pain and anxiety [66].

Further dietary-based interventions under investigation include the use of hyaluronic acid derivatives, such as sodium hyaluronate (SH), for the treatment of constipation-predominant IBS (IBS-C). High-molecular-weight SH has demonstrated effectiveness in improving IBS-C symptoms by reducing pro-inflammatory cytokine levels (IL-1β, IL-18, TNF-α), increasing tight junction protein expressions (claudin-1, occludin, and ZO-1), and restoring intestinal microbial balance across different gut regions [67].

Additionally, recent strategies to maintain intestinal barrier integrity have explored the potential of chitin-glucan, a novel non-digestible dietary fiber derived from Aspergillus niger fungal cell walls [68], as well as newly identified probiotic strains like Lactobacillus gasseri PI41 [69]. These findings highlight the increasing relevance of dietary components in modulating gut health, suggesting that tailored nutritional strategies, including fiber supplementation and probiotic-enriched diets, could play a pivotal role in IBS management.

This study has some limitations. First, relying on a single database (Scopus) for scientific literature searches may compromise the data’s completeness, accuracy, and representativeness. While Scopus is one of the most comprehensive citation databases, it may not fully capture all relevant studies, particularly those indexed exclusively in other databases such as Web of Science (WoS) or PubMed [70]. To ensure a more comprehensive bibliometric analysis, future studies could consider cross-referencing with multiple databases.

However, it is important to note that combining datasets from different databases can introduce challenges such as overlapping records, inconsistent data formats, and technical difficulties in integrating datasets [71]. For instance, most journals are indexed simultaneously in both Scopus and WoS, leading to significant overlap in the publications retrieved using the same search terms [71]. Despite these challenges, cross-referencing with WoS or PubMed could complement Scopus’ strengths and mitigate its individual limitations.

Second, restricting the analysis to English-only articles might exclude significant studies published in other languages. Potential biases could affect the results, such as duration effect bias, which favors older articles, and the misclassification of document types like research letters.

Finally, it is worth pointing out that artificial intelligence (AI) advances will improve a number of tools that could be very useful in planning comparative studies through validation or sensitivity analysis and possibly integrate the PRISMA Artificial Intelligence reporting guidelines for systematic reviews [72].

Despite these limitations, the identified clusters align well with existing knowledge of IBS research.

## 5. Conclusions

This bibliometric analysis provides a comprehensive overview of research trends on intestinal permeability in IBS, emphasizing its complex interactions with gut microbiota and dietary factors. Machine learning tools, such as ASReview, significantly enhanced the efficiency of study selection, ensuring a targeted and comprehensive analysis.

The steady increase in publications reflects the growing interest in this field, driven by increased funding and academic demands. The integration of nutrition-based strategies in managing IBS represents a promising avenue for enhancing clinical outcomes and quality of life for affected individuals. As noted, dietary components such as polyphenols, prebiotics, probiotics, and fiber-rich diets play pivotal roles in modulating intestinal permeability, reducing inflammation, and improving gut barrier function. These interventions can help address some underlying mechanisms implicated in IBS pathophysiology, including dysbiosis, low-grade inflammation, and altered gut motility.

Overall, this analysis provides valuable insights into the current and future research directions in IBS and intestinal permeability, highlighting the critical need for innovative dietary strategies and therapeutic interventions to improve the quality of life for IBS patients.

## Figures and Tables

**Table 1:** Descriptive statistics of articles and citations by year on Irritable Bowel Syndrome and intestinal permeability (*n* = 232).

Year	NP	TC	TC/NP	Percentage (%)
1984	1	111	111.00	0.4
1990	1	64	64.00	0.4
1999	1	129	129.00	0.4
2000	1	88	88.00	0.4
2002	1	361	361.00	0.4
2003	1	171	171.00	0.4
2004	1	248	248.00	0.4
2005	1	20	20.00	0.4
2006	1	406	406.00	0.4
2008	3	588	196.00	1.3
2009	4	955	238.75	1.7
2010	6	777	129.50	2.6
2011	6	343	57.17	2.6
2012	8	665	83.12	3.4
2013	7	562	104.75	3.0
2014	15	1250	83.33	6.5
2015	5	286	57.20	2.2
2016	6	250	41.67	2.6
2017	17	1135	66.76	7.3
2018	15	567	37.80	6.5
2019	19	855	45.00	8.2
2020	21	518	24.67	9.1
2021	26	440	16.92	11.2
2022	24	153	6.38	10.3
2023	26	69	2.65	11.2
2024 ^	15	11	0.73	6.5

NP: total number of publications in that year; TC: Total citations of the document published in that year; TC/NP—Total citation per year on total number of publications in that year; Percentage (%)—the proportion of publications generated in that year relative to the total publication (*n* = 232); ^: Accessed on 10 July 2024.

**Table 2:** Top 10 most globally cited articles on Intestinal Permeability in IBS, evaluated based on titles and abstracts.

SCR	First Author and Year	Title	Journal (IF-2023)	TC	TC/Y
1	Piche T, 2009 [44]	Impaired intestinal barrier integrity in the colon of patients with irritable bowel syndrome: involvement of soluble mediators	Gut (23, Q1)	454	28.38
2	Dunlop SP, 2006 [45]	Abnormal intestinal permeability in subgroups of diarrhea-predominant irritable bowel syndromes	American Journal of Gastroenterology (8, Q1)	406	21.37
3	De Palma G, 2017 [46]	Transplantation of fecal microbiota from patients with irritable bowel syndrome alters gut function and behavior in recipient mice	Science Translational Medicine (15.8, Q1)	374	46.75
4	Tibble JA, 2002 [47]	Use of surrogate markers of inflammation and Rome criteria to distinguish organic from nonorganic intestinal disease	Gastroenterology (25.7, Q1)	361	15.70
5	Zhou Q, 2009 [48]	Intestinal membrane permeability and hypersensitivity in irritable bowel syndrome	Pain (5.9, Q1)	320	20.00
6	Gecse K, 2008 [49]	Increased fecal serine protease activity in diarrhea IBS patients: a colonic luminal factor impairing colonic permeability and sensitivity	Gut (23, Q1)	280	16.47
7	Jalanka-Tuovinen J, 2014 [50]	Fecal microbiota composition and host-microbe cross-talk following gastroenteritis and in post infectious irritable bowel syndrome	Gut (23, Q1)	252	22.91
8	Marshall JK, 2004 [51]	Intestinal permeability in patients with irritable bowel syndrome after a waterborne outbreak of acute gastroenteritis in Walkerton, Ontario	Alimentary Pharmacology & Therapeutics (6.6, Q1)	248	11.81
9	Bertiaux-Vandaële N, 2011 [52]	The expression and the cellular distribution of the tight junction proteins are altered in irritable bowel syndrome patients with differences according to the disease subtype	American Journal of Gastroenterology (8, Q1)	237	16.93
10	Xu D, 2014 [53]	Rifaximin Alters Intestinal Bacteria and Prevents Stress-Induced Gut Inflammation and Visceral Hyperalgesia in Rats	Gastroenterology (25.7, Q1)	233	21.18

Note: SCR = Standard Competition Ranking, TC = Total Citations, TC/Y = Average Citations per Year.

## Data Availability

The data and pipeline used in this study are available on GitHub at https://github.com/MichelangeloAloisio/Bibliometric_Analysis_IBS_permeability/ (accessed on 3 March 2025).

## References

[1] Saha L. (2014). Irritable bowel syndrome: Pathogenesis, diagnosis, treatment, and evidence-based medicine. World J. Gastroenterol..

[2] Tang H.Y., Jiang A.J., Wang X.Y., Wang H., Guan Y.Y., Li F., Shen G.M. (2021). Uncovering the pathophysiology of irritable bowel syndrome by exploring the gut-brain axis: A narrative review. Ann. Transl. Med..

[3] Camilleri M., Lasch K., Zhou W. (2012). Irritable bowel syndrome: Methods, mechanisms, and pathophysiology. The confluence of increased permeability, inflammation, and pain in irritable bowel syndrome. Am. J. Physiol. Gastrointest. Liver Physiol..

[4] Shulman R.J., Jarrett M.E., Cain K.C., Broussard E.K., Heitkemper M.M. (2014). Associations among gut permeability, inflammatory markers, and symptoms in patients with irritable bowel syndrome. J. Gastroenterol..

[5] Li L., Xiong L., Yao J., Zhuang X., Zhang S., Yu Q., Xiao Y., Cui Y., Chen M. (2016). Increased small intestinal permeability and RNA expression profiles of mucosa from terminal ileum in patients with diarrhoea-predominant irritable bowel syndrome. Dig. Liver Dis..

[6] Hanning N., Edwinson A.L., Ceuleers H., Peters S.A., De Man J.G., Hassett L.C., De Winter B.Y., Grover M. (2021). Intestinal barrier dysfunction in irritable bowel syndrome: A systematic review. Therap Adv. Gastroenterol..

[7] Vancamelbeke M., Vermeire S. (2017). The intestinal barrier: A fundamental role in health and disease. Expert. Rev. Gastroenterol. Hepatol..

[8] Horowitz A., Chanez-Paredes S.D., Haest X., Turner J.R. (2023). Paracellular permeability and tight junction regulation in gut health and disease. Nat. Rev. Gastroenterol. Hepatol..

[9] Gunzel D., Yu A.S. (2013). Claudins and the modulation of tight junction permeability. Physiol. Rev..

[10] Kuo W.T., Odenwald M.A., Turner J.R., Zuo L. (2022). Tight junction proteins occludin and ZO-1 as regulators of epithelial proliferation and survival. Ann. N. Y. Acad. Sci..

[11] Camilleri M. (2019). Leaky gut: Mechanisms, measurement and clinical implications in humans. Gut.

[12] Di Vincenzo F., Del Gaudio A., Petito V., Lopetuso L.R., Scaldaferri F. (2024). Gut microbiota, intestinal permeability, and systemic inflammation: A narrative review. Intern. Emerg. Med..

[13] Ringel Y., Maharshak N. (2013). Intestinal microbiota and immune function in the pathogenesis of irritable bowel syndrome. Am. J. Physiol. Gastrointest. Liver Physiol..

[14] Huang C., Song P., Fan P., Hou C., Thacker P., Ma X. (2015). Dietary Sodium Butyrate Decreases Postweaning Diarrhea by Modulating Intestinal Permeability and Changing the Bacterial Communities in Weaned Piglets. J. Nutr..

[15] Khoshbin K., Camilleri M. (2020). Effects of dietary components on intestinal permeability in health and disease. Am. J. Physiol. Gastrointest. Liver Physiol..

[16] Inczefi O., Bacsur P., Resal T., Keresztes C., Molnar T. (2022). The Influence of Nutrition on Intestinal Permeability and the Microbiome in Health and Disease. Front. Nutr..

[17] Nogal A., Valdes A.M., Menni C. (2021). The role of short-chain fatty acids in the interplay between gut microbiota and diet in cardio-metabolic health. Gut Microbes.

[18] Linsalata M., Riezzo G., Clemente C., D’Attoma B., Russo F. (2020). Noninvasive Biomarkers of Gut Barrier Function in Patients Suffering from Diarrhea Predominant-IBS: An Update. Dis. Markers.

[19] Russo F., Chimienti G., Riezzo G., Linsalata M., D’Attoma B., Clemente C., Orlando A. (2018). Adipose Tissue-Derived Biomarkers of Intestinal Barrier Functions for the Characterization of Diarrhoea-Predominant IBS. Dis. Markers.

[20] Linsalata M., Riezzo G., D’Attoma B., Clemente C., Orlando A., Russo F. (2018). Noninvasive biomarkers of gut barrier function identify two subtypes of patients suffering from diarrhoea predominant-IBS: A case-control study. BMC Gastroenterol..

[21] Prospero L., Riezzo G., Linsalata M., Orlando A., D’Attoma B., Di Masi M., Martulli M., Russo F. (2021). Somatization in patients with predominant diarrhoea irritable bowel syndrome: The role of the intestinal barrier function and integrity. BMC Gastroenterol..

[22] Manoj Kumar L., George R.J., S A.P. (2023). Bibliometric Analysis for Medical Research. Indian. J. Psychol. Med..

[23] Stefanis C., Stavropoulou E., Giorgi E., Voidarou C.C., Constantinidis T.C., Vrioni G., Tsakris A. (2023). Honey’s Antioxidant and Antimicrobial Properties: A Bibliometric Study. Antioxidants.

[24] Stefanis C., Giorgi E., Tselemponis G., Voidarou C., Skoufos I., Tzora A., Tsigalou C., Kourkoutas Y., Constantinidis T.C., Bezirtzoglou E. (2023). Terroir in View of Bibliometrics. Stats.

[25] Aria M., Cuccurullo C. (2017). bibliometrix: An R-tool for comprehensive science mapping analysis. J. Informetr..

[26] van Eck N., Waltman L. (2010). Software survey: VOSviewer, a computer program for bibliometric mapping. Scientometrics.

[27] van de Schoot R., de Bruin J., Schram R., Zahedi P., de Boer J., Weijdema F., Kramer B., Huijts M., Hoogerwerf M., Ferdinands G. (2021). An open source machine learning framework for efficient and transparent systematic reviews. Nat. Mach. Intell..

[28] Pranckutė R. (2021). Web of Science (WoS) and Scopus: The Titans of Bibliographic Information in Today’s Academic World. Publications.

[29] Infantino V., Riva A., Petrangolini G., Allegrini P., Perna S., Iannello G., Peroni G., Gasparri C., Rondanelli M. (2021). The Use of Berberine in Diabetes and Metabolic Syndrome: Two Sides of the Same Coin. A Bibliometric Analysis. Curr. Nutr. Food Sci..

[30] Morandi G., Guido D., Tagliabue A. (2015). A bibliometric study of scientific literature on the dietary therapies for epilepsy in Scopus. Nutr. Neurosci..

[31] Rondanelli M., Perna S., Peroni G., Guido D. (2016). A bibliometric study of scientific literature in Scopus on botanicals for treatment of androgenetic alopecia. J. Cosmet. Dermatol..

[32] Baas J., Schotten M., Plume A., Côté G., Karimi R. (2020). Scopus as a curated, high-quality bibliometric data source for academic research in quantitative science studies. Quant. Sci. Stud..

[33] Awad K., Barmeyer C., Bojarski C., Nagel O., Lee I.M., Schweiger M.R., Schulzke J.D., Bucker R. (2023). Impaired Intestinal Permeability of Tricellular Tight Junctions in Patients with Irritable Bowel Syndrome with Mixed Bowel Habits (IBS-M). Cells.

[34] Linsalata M., Riezzo G., Orlando A., D’Attoma B., Prospero L., Ignazzi A., Losurdo G., Di Leo A., Giannelli G., Russo F. (2023). The Role of Intestinal Barrier Function in Overweight Patients with IBS with Diarrhea Undergoing a Long-Term Low Fermentable Oligo-, Di-, and Monosaccharide and Polyol Diet. Nutrients.

[35] Awad K., Barmeyer C., Bojarski C., Nagel O., Lee I.M., Schweiger M.R., Schulzke J.D., Bucker R. (2023). Epithelial Barrier Dysfunction in Diarrhea-Predominant Irritable Bowel Syndrome (IBS-D) via Downregulation of Claudin-1. Cells.

[36] Filippone A., Ardizzone A., Bova V., Lanza M., Casili G., Cuzzocrea S., Esposito E., Campolo M., Paterniti I. (2022). A Combination of Xyloglucan, Pea Protein and Chia Seed Ameliorates Intestinal Barrier Integrity and Mucosa Functionality in a Rat Model of Constipation-Predominant Irritable Bowel Syndrome. J. Clin. Med..

[37] Ceren Akgor M., Vuralli D., Sucu D.H., Gokce S., Tasdelen B., Gultekin F., Bolay H. (2023). Distinct Food Triggers for Migraine, Medication Overuse Headache and Irritable Bowel Syndrome. J. Clin. Med..

[38] Ammar R.M., Pferschy-Wenzig E.M., Van den Abbeele P., Verstrepen L., Ghyselinck J., Thumann T., Bauer R. (2023). Possible role of the gut microbiome in mediating the beneficial effects of the six-herbal formulation STW 5-II on digestive health. Phytomedicine.

[39] van Orten-Luiten A.B., de Roos N.M., Majait S., Witteman B.J.M., Witkamp R.F. (2022). Effects of Cannabidiol Chewing Gum on Perceived Pain and Well-Being of Irritable Bowel Syndrome Patients: A Placebo-Controlled Crossover Exploratory Intervention Study with Symptom-Driven Dosing. Cannabis Cannabinoid Res..

[40] Zimmermann J., Longin F.H., Schweinlin A., Basrai M., Bischoff S.C. (2022). No Difference in Tolerance between Wheat and Spelt Bread in Patients with Suspected Non-Celiac Wheat Sensitivity. Nutrients.

[41] van Dijk S.H.B., Brusse-Keizer M.G.J., Bucsan C.C., van der Palen J., Doggen C.J.M., Lenferink A. (2023). Artificial intelligence in systematic reviews: Promising when appropriately used. BMJ Open.

[42] Ferdinands G., Schram R., de Bruin J., Bagheri A., Oberski D.L., Tummers L., Teijema J.J., van de Schoot R. (2023). Performance of active learning models for screening prioritization in systematic reviews: A simulation study into the Average Time to Discover relevant records. Syst. Rev..

[43] Park Y.S., Kang S.B., Marchelletta R.R., Penrose H.M., Ruiter-Visser R., Jung B., Docherty M.J., Boland B.S., Sandborn W.J., McCole D.F. (2023). The ClC-2 Chloride Channel Activator, Lubiprostone, Improves Intestinal Barrier Function in Biopsies from Crohn’s Disease but Not Ulcerative Colitis Patients. Pharmaceutics.

[44] Piche T., Barbara G., Aubert P., Bruley des Varannes S., Dainese R., Nano J.L., Cremon C., Stanghellini V., De Giorgio R., Galmiche J.P. (2009). Impaired intestinal barrier integrity in the colon of patients with irritable bowel syndrome: Involvement of soluble mediators. Gut.

[45] Dunlop S.P., Hebden J., Campbell E., Naesdal J., Olbe L., Perkins A.C., Spiller R.C. (2006). Abnormal intestinal permeability in subgroups of diarrhea-predominant irritable bowel syndromes. Am. J. Gastroenterol..

[46] De Palma G., Lynch M.D., Lu J., Dang V.T., Deng Y., Jury J., Umeh G., Miranda P.M., Pigrau Pastor M., Sidani S. (2017). Transplantation of fecal microbiota from patients with irritable bowel syndrome alters gut function and behavior in recipient mice. Sci. Transl. Med..

[47] Tibble J.A., Sigthorsson G., Foster R., Forgacs I., Bjarnason I. (2002). Use of surrogate markers of inflammation and Rome criteria to distinguish organic from nonorganic intestinal disease. Gastroenterology.

[48] Zhou Q., Zhang B., Verne G.N. (2009). Intestinal membrane permeability and hypersensitivity in the irritable bowel syndrome. Pain..

[49] Gecse K., Roka R., Ferrier L., Leveque M., Eutamene H., Cartier C., Ait-Belgnaoui A., Rosztoczy A., Izbeki F., Fioramonti J. (2008). Increased faecal serine protease activity in diarrhoeic IBS patients: A colonic lumenal factor impairing colonic permeability and sensitivity. Gut.

[50] Jalanka-Tuovinen J., Salojarvi J., Salonen A., Immonen O., Garsed K., Kelly F.M., Zaitoun A., Palva A., Spiller R.C., de Vos W.M. (2014). Faecal microbiota composition and host-microbe cross-talk following gastroenteritis and in postinfectious irritable bowel syndrome. Gut.

[51] Marshall J.K., Thabane M., Garg A.X., Clark W., Meddings J., Collins S.M., Investigators W.E.L. (2004). Intestinal permeability in patients with irritable bowel syndrome after a waterborne outbreak of acute gastroenteritis in Walkerton, Ontario. Aliment. Pharmacol. Ther..

[52] Bertiaux-Vandaele N., Youmba S.B., Belmonte L., Lecleire S., Antonietti M., Gourcerol G., Leroi A.M., Dechelotte P., Menard J.F., Ducrotte P. (2011). The expression and the cellular distribution of the tight junction proteins are altered in irritable bowel syndrome patients with differences according to the disease subtype. Am. J. Gastroenterol..

[53] Xu D., Gao J., Gillilland M., Wu X., Song I., Kao J.Y., Owyang C. (2014). Rifaximin alters intestinal bacteria and prevents stress-induced gut inflammation and visceral hyperalgesia in rats. Gastroenterology.

[54] Liu T., Zhang K. (2023). Effects of the digital economy on carbon emissions in China: An analysis based on different innovation paths. Environ. Sci. Pollut. Res. Int..

[55] Groschwitz K.R., Hogan S.P. (2009). Intestinal barrier function: Molecular regulation and disease pathogenesis. J. Allergy Clin. Immunol..

[56] Ji J., Jin W., Liu S.J., Jiao Z., Li X. (2023). Probiotics, prebiotics, and postbiotics in health and disease. MedComm.

[57] Raskov H., Burcharth J., Pommergaard H.C., Rosenberg J. (2016). Irritable bowel syndrome, the microbiota and the gut-brain axis. Gut Microbes.

[58] Li D., Yu S., Long Y., Shi A., Deng J., Ma Y., Wen J., Li X., Liu S., Zhang Y. (2022). Tryptophan metabolism: Mechanism-oriented therapy for neurological and psychiatric disorders. Front. Immunol..

[59] Fortea M., Albert-Bayo M., Abril-Gil M., Ganda Mall J.P., Serra-Ruiz X., Henao-Paez A., Exposito E., Gonzalez-Castro A.M., Guagnozzi D., Lobo B. (2021). Present and Future Therapeutic Approaches to Barrier Dysfunction. Front. Nutr..

[60] Liu Y.L., Liu J.S. (2021). Irritable bowel syndrome in China: A review on the epidemiology, diagnosis, and management. Chin. Med. J..

[61] Hungin A.P., Chang L., Locke G.R., Dennis E.H., Barghout V. (2005). Irritable bowel syndrome in the United States: Prevalence, symptom patterns and impact. Aliment. Pharmacol. Ther..

[62] Arie H., Nozu T., Miyagishi S., Ida M., Izumo T., Shibata H. (2019). Grape Seed Extract Eliminates Visceral Allodynia and Colonic Hyperpermeability Induced by Repeated Water Avoidance Stress in Rats. Nutrients.

[63] Maqoud F., Orlando A., Tricarico D., Antonacci M., Di Turi A., Giannelli G., Russo F. (2024). Anti-Inflammatory Effects of a Novel Acetonitrile-Water Extract of Lens Culinaris against LPS-Induced Damage in Caco-2 Cells. Int. J. Mol. Sci..

[64] Russo F., Riezzo G., Linsalata M., Orlando A., Tutino V., Prospero L., D’Attoma B., Giannelli G. (2022). Managing Symptom Profile of IBS-D Patients With Tritordeum-Based Foods: Results From a Pilot Study. Front. Nutr..

[65] Linsalata M., Riezzo G., Orlando A., D’Attoma B., Prospero L., Tutino V., Notarnicola M., Russo F. (2021). The Relationship between Low Serum Vitamin D Levels and Altered Intestinal Barrier Function in Patients with IBS Diarrhoea Undergoing a Long-Term Low-FODMAP Diet: Novel Observations from a Clinical Trial. Nutrients.

[66] Meynier M., Daugey V., Mallaret G., Gervason S., Meleine M., Barbier J., Aissouni Y., Lolignier S., Bonnet M., Ardid D. (2024). Pasteurized akkermansia muciniphila improves irritable bowel syndrome-like symptoms and related behavioral disorders in mice. Gut Microbes.

[67] Cui L., Zou S., Liu J., Lv H., Li H., Zhang Z. (2024). Potential effects of sodium hyaluronate on constipation-predominant irritable bowel syndrome. Int. Immunopharmacol..

[68] Valibouze C., Dubuquoy C., Chavatte P., Genin M., Maquet V., Modica S., Desreumaux P., Rousseaux C. (2024). Chitin-glucan improves important pathophysiological features of irritable bowel syndrome. World J. Gastroenterol..

[69] Torres-Maravilla E., Carvalho F.A., Holowacz S., Delannoy J., Lenoir L., Jacouton E., Barbut F., Langella P., Bermudez-Humaran L.G., Waligora-Dupriet A.J. (2024). Screening of probiotic strains to improve visceral hypersensitivity in irritable bowel syndrome by using in vitro and in vivo approaches. Benef. Microbes.

[70] Zhu J., Liu W. (2020). A tale of two databases: The use of Web of Science and Scopus in academic papers. Scientometrics.

[71] Öztürk O., Kocaman R., Kanbach D.K. (2024). How to design bibliometric research: An overview and a framework proposal. Rev. Manag. Sci..

[72] Cacciamani G.E., Chu T.N., Sanford D.I., Abreu A., Duddalwar V., Oberai A., Kuo C.C.J., Liu X., Denniston A.K., Vasey B. (2023). PRISMA AI reporting guidelines for systematic reviews and meta-analyses on AI in healthcare. Nat. Med..

